# Prediction of post-stroke cognitive impairment by Montreal Cognitive Assessment (MoCA) performances in acute stroke: comparison of three normative datasets

**DOI:** 10.1007/s40520-022-02133-9

**Published:** 2022-04-20

**Authors:** Emilia Salvadori, Ilaria Cova, Francesco Mele, Simone Pomati, Leonardo Pantoni

**Affiliations:** 1 NEUROFARBA Department, Neuroscience Section, University of Florence, Florence, Italy; 2 Neurology Unit, Luigi Sacco University Hospital, Milan, Italy; 3 Stroke and Dementia Lab, “Luigi Sacco” Department of Biomedical and Clinical Sciences, University of Milan, Milan, Italy

**Keywords:** Acute stroke, Neuropsychology, Montreal Cognitive Assessment, Post-stroke cognitive impairment, Normality cut-off

## Abstract

**Background:**

Cognitive assessment in acute stroke is relevant for identifying patients at risk of persistent post-stroke cognitive impairment (PSCI). Despite preliminary evidence on MoCA accuracy, there is no consensus on its optimal score in the acute stroke setting to predict PSCI.

**Aims:**

(1) To explore whether the application of different normative datasets to MoCA scores obtained in the acute stroke setting results in variable frequency of patients defined as cognitively impaired; (2) to assess whether the normality cut-offs provided by three normative datasets predict PSCI at 6–9 months; (3) to calculate alternative MoCA cut-offs able to predict PSCI.

**Methods:**

Consecutive stroke patients were reassessed at 6–9 months with extensive neuropsychological and functional batteries for PSCI determination.

**Results:**

Out of 207 enrolled patients, 118 (57%) were followed-up (mean 7.4 ± 1.7 months), and 77 of them (65%) received a PSCI diagnosis. The application of the normality thresholds provided by the 3 normative datasets yielded to variable (from 28.5% to 41%) rates of patients having an impaired MoCA performance, and to an inadequate accuracy in predicting PSCI, maximizing specificity instead of sensitivity. In ROC analyses, a MoCA score of 22.82, adjusted according to the most recent normative dataset, achieved a good diagnostic accuracy in predicting PSCI.

**Conclusions:**

The classification of acute stroke patients as normal/impaired based on MoCA thresholds proposed by general population normative datasets underestimated patients at risk of persistent PSCI. We calculated a new adjusted MoCA score predictive of PSCI in acute stroke patients to be further tested in larger studies.

## Introduction

Post-stroke cognitive impairment (PSCI) encompasses all forms and degrees of cognitive disorders whose onset is temporally related with a stroke [1, 2]. The cognitive profile of PSCI is heterogeneous and may include deficits in cortical functions (e.g., aphasia, neglect, apraxia, agnosia) as well as a dysexecutive syndrome caused by the dysfunction of integrated brain networks [3–6]. In the acute phase, approximately 75% of stroke patients experience cognitive deficits [7–9]. In the chronic phase, cognitive impairment persists in approximately 50% of patients and is associated with poor functional and survival outcomes [2, 9, 10].

The identification of patients at risk of persistent PSCI in the acute phase might help clinicians to early plan treatment options as well as serial cognitive assessments. Because cognitive assessment in acute stroke must fulfill a feasibility criterion imposed by the setting and patients’ conditions, a multidomain, quick and easy to use, screening tool represents the gold standard. Diagnostic accuracy of such a tool would have to reach a sensitivity ≥ 80% and a specificity ≥ 60% and should be evaluated with respect to a long-term PSCI diagnosis based on a comprehensive neuropsychological test battery [8, 11].

During the last decade, several studies have tested Montreal Cognitive Assessment (MoCA) in acute stroke patients, and some encouraging evidence on its accuracy and predictive validity have made it one of the best candidates in this setting [12–15]. However, one unsolved issue in the use of MoCA in acute stroke patients concerns the definition of cut-offs able to predict patients at risk of persistent PSCI. In this peculiar setting, MoCA scores predictive of PSCI were variable across studies evaluating acute stroke patients, ranging from 19 to 22, and were based on raw scores [15, 16]. The latter represent a relevant issue because MoCA normative studies have clearly showed the wide impact of demographic and cultural factors on its performance [17].

At present, three general population normative datasets are available for the Italian version of the MoCA. Normative values were firstly published by Conti and colleagues in 2004 (225 subjects, age range 60–80), then by Santangelo and colleagues in 2005 (415 subjects, age range 21–95), and recently by Aiello and colleagues in 2021 (579 subjects, age range 21–96) [18–20]. The use of demographically and culturally appropriate correction norms for the evaluation of MoCA performances in acute stroke might represent an added value on the way of defining predictive scores in this setting.

The aims of this study were: (1) to explore whether the application of different normative datasets to the MoCA scores obtained in the acute stroke setting results in variable frequency of patients defined as cognitively impaired; (2) to assess whether the normality cut-offs provided by each of the three normative datasets predict PSCI at 6–9 months; (3) to calculate alternative cut-offs of MoCA performances obtained in the acute phase able to predict PSCI.

## Methods

The present study is based on data collected in two previous studies carried out at the stroke units of the Luigi Sacco University Hospital (Milan) and of the Careggi University Hospital (Florence), Italy [21, 22].

Inclusion criteria were diagnosis of stroke (ischemic or hemorrhagic) or transient ischemic attack and age > 18 years. No exclusion criterion was applied. Informed consent was obtained by patients or caregivers.

During stroke unit hospitalization, patients were evaluated at bedside by means of the MoCA, and the National Institute of Health Stroke Scale (NIHSS) was used to estimate index stroke severity [23]. MoCA performance was evaluated using the corrections norms reported in the three normative datasets [18–20]. Demographically adjusted scores have been calculated based on the following regression equations extracted by normative studies:

- Conti [18]
  $${\text{adjusted}}\;{\text{score}} = {\text{raw}}\;{\text{score}} + 0.175*\left( {{\text{age}} - 70.08} \right) + 24.3*\left( {\frac{1}{{{\text{years}}\;{\text{of}}\;{\text{education}}}} - 0.126} \right)$$
- Santangelo [19]
  $${\text{adjusted}}\;{\text{score}} = {\text{raw}}\;{\text{score}} - 4.228*\left[ {\log_{10} \left( {100 - {\text{age}}} \right) - 1.58} \right] - 3.201*\left( {\sqrt {{\text{years}}\;{\text{of}}\;{\text{education}}} - 3.25} \right)$$
- Aiello [20]
  $${\text{adjusted}}\;{\text{score}} = {\text{raw}}\;{\text{ score + 0}}.000008*\left( {{\text{age}}^{3} - 297697.184801} \right) - 3.331407*\left[ {\ln \left( {{\text{years}}\;{\text{of}}\;{\text{education}}} \right) - 2.325648} \right]$$

All normative studies applied an equivalent score (ES) methodology that is a non-parametric (percentiles based) norming method that allows to convert age and education adjusted scores into an ordinal 5-point scale [24]. Definitions of ES classification and cut-offs of the normative studies were as follows: ES = 0, impaired performance (a demographically adjusted score below the outer confidence limit for the 5th centile of the normal population), corresponded to a cut-off of 18.58 in Aiello, 17.36 in Conti, and 15.50 in Santangelo normative datasets; ES = 1, borderline performance; ES = 2, 3 and 4, normal performance [18–20].

Between 6 and 9 months after the acute event, each patient was contacted to undergo an extensive neuropsychological and functional evaluation. Neuropsychological tests used at the follow-up examination in the two centers are shown in Table 1. Functional status was measured by means of the modified Rankin Scale (mRS) [35], ADL and IADL scales [36, 37].

**Table 1:** Neuropsychological tests used at the follow-up examination

Cognitive domain	Luigi Sacco University Hospital	Careggi University Hospital
Memory	Rey Auditory-Verbal Learning Test [25]	
	Rey figure test—delayed recall [26]	
Attention and executive functions		Visual search test [27]
	Symbol digit modalities test [28]	
	Trail making test (TMT, part A and B) [29]	
	Color word Stroop test [30]	
Language	Phonemic and semantic verbal fluency tasks [31, 32]	
	Phrase construction test [25]	
	Neuropsychological examination for aphasia [33]	
Visuospatial functions	Rey figure test—copy [26]	
	Apple test [34]	

The study outcome was PSCI diagnosis, including both Mild Cognitive Impairment (MCI) and dementia. Cognitive impairment was diagnosed based on the presence of an impaired performance in at least one test (a demographically adjusted score below the 5th centile of the normal population). The differential diagnosis between MCI and dementia was based on the presence of a functional dependence in ADL or IADL scales. Specifically, dementia was diagnosed only if the functional impairment was not a motor/sensory sequelae of the cerebrovascular event, nor a consequence of other diseases or physical limitations.

### Statistical analysis

Descriptive statistics were used to describe the study cohort in terms of baseline demographic and clinical characteristics. To verify for a selection bias, bivariate statistical analyses (independent samples *t*-tests, Chi-square tests) were used to compare baseline characteristics between patients with or without follow-up.

For the statistical analyses purposes, MoCA performance was analyzed either as demographically adjusted scores (continuous), ES distribution (categorical), or impaired (ES = 0) vs. normal (ES = 1–4) (dichotomic).

Bivariate statistical analyses (independent samples t-tests, ANOVAs) were used to compare MoCA demographically adjusted scores between patients with or without PSCI at follow-up, as well as across cognitively normal, MCI and demented patients. Logistic regression models were further applied to evaluate if baseline MoCA adjusted scores were associated with the risk of PSCI at follow-up. Chi-square tests and accuracy indexes (sensitivity, specificity, positive and negative predictive values) were calculated for the normality thresholds of each normative study.

Receiver operator characteristic (ROC) analysis was used to examine the ability of the demographically adjusted MoCA scores to distinguish between patients with or without PSCI at follow-up. For each normative study, area under the curve, sensitivity, specificity, positive and negative predictive values were calculated to identify an optimal predictive score.

All data analyses were performed using SPSS 27 and a significance threshold set at *p* < 0.05.

## Results

Two hundred and seven patients (mean age 76 ± 9.6 years, mean education 9.1 ± 4.5 years, females 34%) were recruited during their hospitalization in stroke unit (*n* = 82 Careggi University Hospital, *n* = 125 Luigi Sacco University Hospital).

**Fig. 1:**
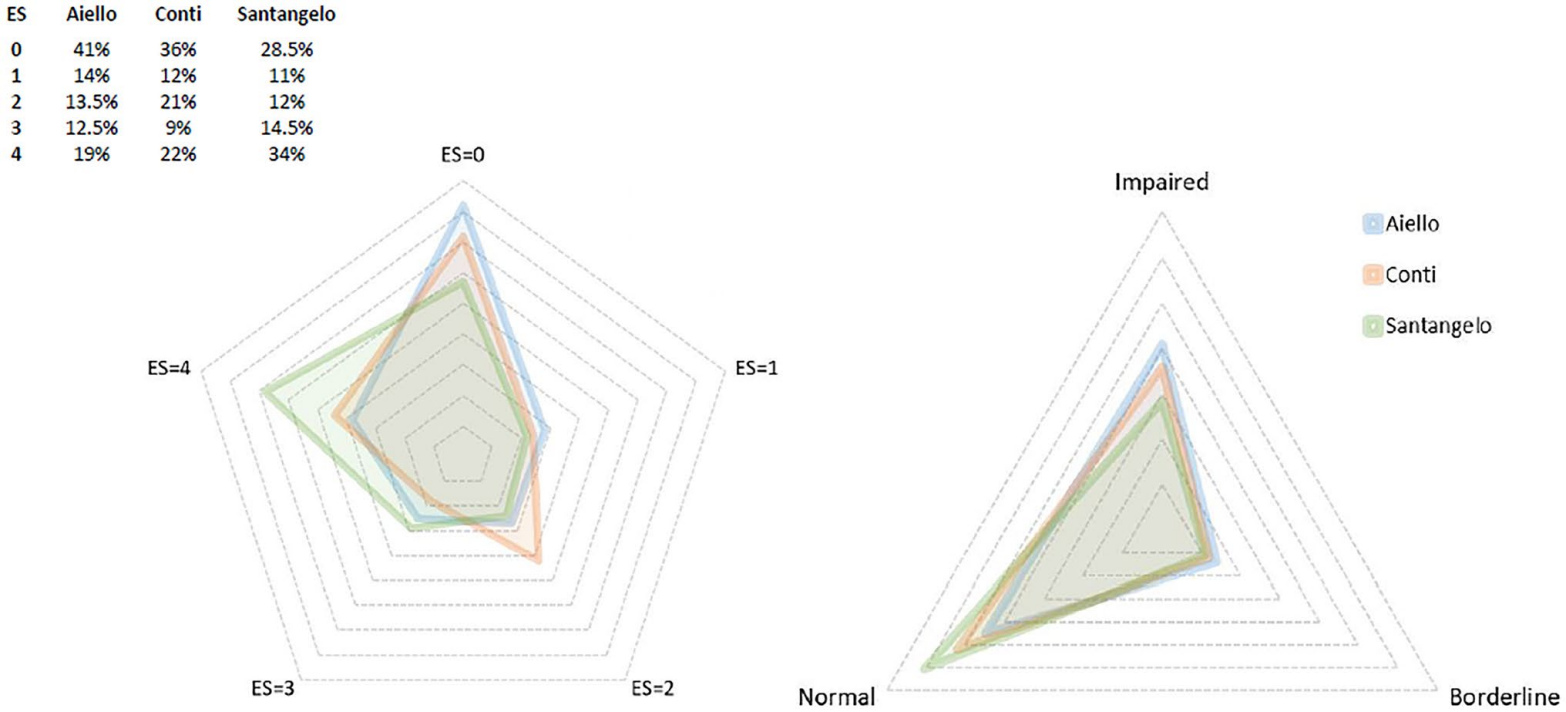
Radar charts showing baseline Montreal Cognitive Assessment (MoCA) performances evaluated by means of Conti, Santangelo, and Aiello normative datasets. Left panel: equivalent scores (ES) distributions. Right panel: MoCA performances categorized as impaired (ES = 0), borderline (ES = 1), or normal (ES ≥ 2)

Applying the different normality thresholds of each of the 3 normative datasets, rates of patients having an impaired baseline MoCA performance ranged from 28.5% using Santangelo’s, to 36% using Conti’s, and 41% using Aiello’s normative datasets (Fig. 1).

Out of the 207 enrolled patients, 118 (57%) were evaluated at follow-up (mean follow-up time 7.4 ± 1.7 months). As reported in Table 2, compared to patients with a follow-up evaluation, drop-outs were significantly older and had a worse MoCA performance at baseline.

**Table 2:** Demographic and clinical characteristics of the baseline cohort, and comparisons between patients who completed the study and drop-outs

	Baseline cohort *n* = 207	Follow-up cohort *n* = 118	Drop-outs *n* = 89	*p*
Age (years)	76 ± 9.6	**74.7 ± 8.9**	**77.7 ± 10.3**	**0.031**
Sex (female)	70 (34%)	38 (32%)	32 (36%)	0.572
Education (years)	9.1 ± 4.5	9.1 ± 4.6	9.1 ± 4.4	0.966
Type of cerebrovascular event				
TIA	13 (6%)	7 (6%)	6 (7%)	
Ischemic stroke	174 (84%)	99 (84%)	75 (84%)	0.938
Hemorrhagic stroke	20 (10%)	12 (10%)	8 (9%)	
Stroke severity (NIHSS score)	2.1 ± 3.1	1.9 ± 2.9	2.4 ± 3.3	0.345
MoCA (raw total score)	17.1 ± 6.9	**18.1 ± 6.8**	**15.8 ± 7.1**	**0.021**
MoCA impaired performance				
Aiello	85 (41%)	**38 (32%)**	**47 (53%)**	**0.002**
Conti	75 (36%)	**33 (28%)**	**42 (47%)**	**0.004**
Santagelo	59 (28.5)	28 (24%)	31 (35%)	0.080

Bold indicates statistically significant differences between the groups

*MoCA* Montreal Cognitive Assessment; *NIHSS* National Institute of Health Stroke Scale; *TIA* transient ischemic attack

Out of the 118 followed-up patients, 77 (65%) received a PSCI diagnosis (55 MCI and 22 dementia). No statistically significant differences were found in rates of patients diagnosed as PSCI between Careggi and Luigi Sacco University Hospitals (69% vs. 61%, respectively, *p* = 0.348).

Applying all the three normative studies, baseline MoCA adjusted scores were significantly different between patients with or without PSCI, and across the three categories of cognitively normal, MCI and demented patients (Table 3). For all the three normative datasets, the logistic regression models showed that the loss of one point on baseline MoCA increased the risk of PSCI of approximately 30%, specifically: Conti OR = 1.29 (95%CI 1.15–1.45), Santangelo OR = 1.37 (95%CI 1.19–1.58), Aiello OR = 1.39 (95%CI 1.21–1.59).

**Table 3:** Baseline Montreal Cognitive Assessment (MoCA) adjusted scores (mean ± SD): comparison across patients with or without post-stroke cognitive impairment (PSCI) at follow-up

	No PSCI *n* = 41	PSCI *n* = 77	p*	MCI *n* = 55	Dementia *n* = 22	*p*^#^
Aiello	23.9 ± 3.2	17.9 ± 5.5	0.001	19.2 ± 5	14.8 ± 5.3	0.001
Conti	22.9 ± 3.3	17.5 ± 5.7	0.001	18.5 ± 5.1	14.9 ± 6.4	0.001
Santangelo	23.9 ± 3.2	17.9 ± 5.6	0.001	19.3 ± 5.1	14.6 ± 5.4	0.001

*MCI* mild cognitive impairment

^*^Comparisons between patients with or without PSCI: independent samples *t* tests

^*#*^Comparisons across no PSCI, MCI and demented patients: ANOVAs

However, rates of patients that had a normal MoCA performance in the acute phase according to normative datasets but received at follow-up a PSCI diagnosis ranged from 51% using Aiello’s, to 54% using Conti’s and 55% using Santangelo’s dataset. Accuracy indexes (Table 4) showed that the application of the normality thresholds maximized specificity (ranging from 95% to 98%) in respect of sensitivity (ranging from 35% to 47%); none of the normative studies achieved an adequate level of diagnostic accuracy (i.e., sensitivity ≥ 80%, specificity ≥ 60%).

**Table 4:** Accuracy of the baseline Montreal Cognitive Assessment (MoCA) performance evaluated according to normality thresholds proposed by normative datasets on the post-stroke cognitive impairment (PSCI) diagnosis at follow-up

	Baseline MoCA performance	No PSCI *n* = 41	PSCI *n* = 77	*p*	SE	SP	PPV	NPV
Aiello	Impaired	2 (5%)	36 (47%)	0.001	47%	95%	95%	49%
	Normal	39 (95%)	41 (53%)					
Conti	Impaired	2 (5%)	31 (40%)	0.001	40%	95%	94%	46%
	Normal	39 (95%)	46 (60%)					
Santangelo	Impaired	1 (2%)	27 (35%)	0.001	35%	98%	96%	44%
	Normal	40 (98%)	50 (65%)					

Impaired baseline MoCA performance corresponded to an equivalent score = 0

Normal baseline MoCA performance corresponded to an equivalent score ≥ 1

*SE* sensitivity; *SP* specificity; *PPV* positive predictive value; *NPV* negative predictive value

**Fig. 2:**
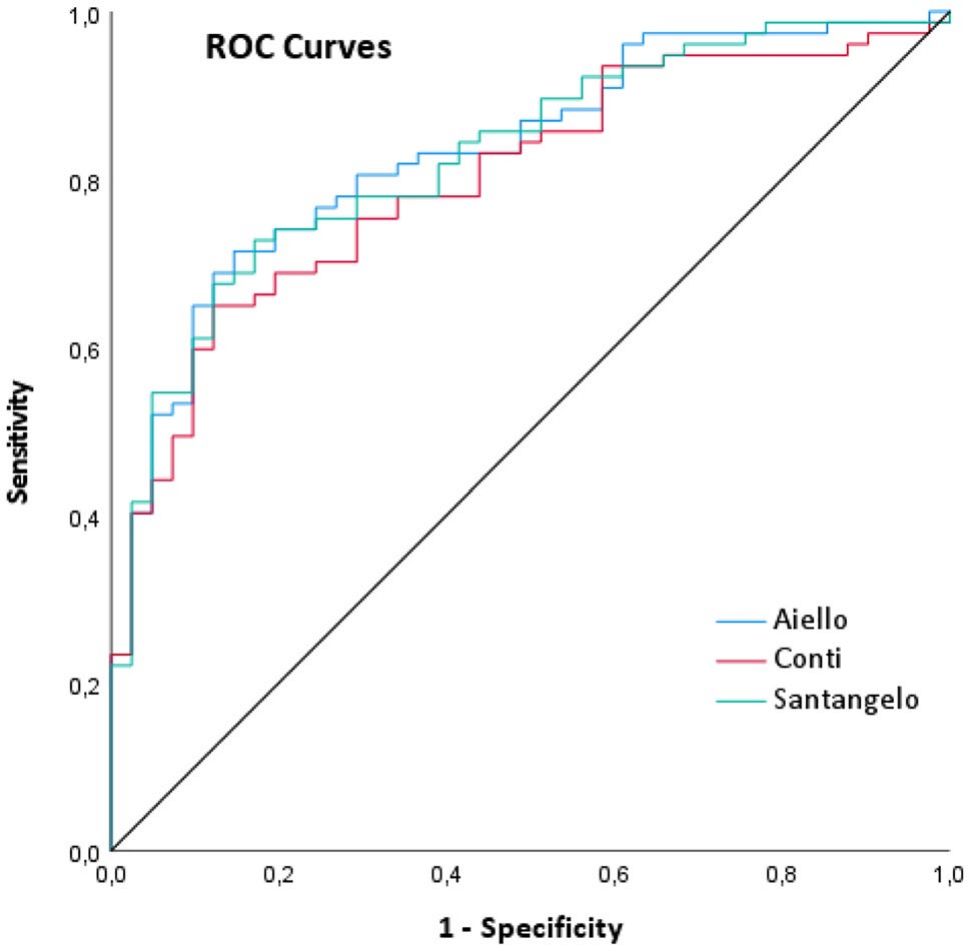
ROC curves showing sensibility and specificity of Montreal Cognitive Assessment (MoCA) scores demographically adjusted according to Conti, Santangelo and Aiello normative datasets (independent variables) for the identification of post stroke cognitive impairment at follow-up (dependent variable)

To identify a demographically adjusted MoCA score able to detect patients at risk of PSCI, ROC analyses were conducted separately for the 3 normative studies. All areas under the curves (Conti = 0.800, Santangelo = 0.828, Aiello = 0.833) were statistically significant at *p* < 0.001 (Fig. 2). As shown in Table 5, an Aiello-adjusted MoCA score of 22.82 achieved a good diagnostic accuracy, i.e., sensitivity 81%, specificity 71%, positive predictive value 84%, negative predictive value 66%. The only other potential predictive score that achieved an adequate diagnostic accuracy (sensitivity 82%, specificity 61%, positive predictive value 80%, negative predictive value 64%) was a Santangelo-adjusted MoCA score of 23.45.

**Table 5:** Sensitivity and specificity of Montreal Cognitive Assessment (MoCA) scores demographically adjusted according to normative datasets for the identification of Post Stroke Cognitive Impairment (PSCI) at follow-up

Aiello			Conti			Santangelo		
Cut-off	Sensitivity	Specificity	Cut-off	Sensitivity	Specificity	Cut-off	Sensitivity	Specificity
21.16	70	85	20.73	70	76	21.41	70	83
21.20	71	85	20.86	70	73	21.53	71	83
21.26	71	83	20.92	70	71	21.64	73	83
21.38	71	81	20.95	71	71	21.69	73	81
21.54	73	81	20.99	73	71	21.75	74	81
21.74	74	81	21.13	74	71	21.86	74	78
21.99	74	78	21.28	75	71	22.06	74	76
22.17	74	76	21.33	75	68	22.34	75	76
22.28	75	76	21.42	75	66	22.50	75	73
22.40	77	76	21.55	77	66	22.63	75	71
22.48	77	73	21.64	78	66	22.75	77	71
22.54	78	73	21.73	78	63	22.79	78	71
22.58	78	71	21.97	78	61	22.86	78	68
22.68	79	71	22.24	78	59	22.91	78	66
**22.82**	**81**	**71**	22.35	78	56	23.14	78	63
22.92	81	68	22.41	79	56	23.37	78	61
22.97	81	66	22.50	81	56	23.40	79	61
23.05	82	66	22.59	82	56	23.44	81	61
23.10	82	63	22.64	83	56	**23.45**	**82**	**61**
23.18	83	63	22.90	83	54	23.50	82	59
23.30	83	61	23.16	83	51	23.56	83	59
23.40	83	59	23.19	84	51	23.58	84	59
23.45	83	56	23.23	84	49	23.61	84	56
23.64	83	54				23.66	86	56
23.81	83	51				23.86	86	54
23.83	84	51				24.07	86	51
23.90	86	51				24.19	86	49
23.98	87	51						
24.06	87	49						

Bold indicates the optimal cut-offs

## Discussion

The present study represents an effort to examine and compare the use of different normative datasets, and thus of different general population normality thresholds, in the evaluation of MoCA performances in acute stroke and to evaluate the accuracy of these thresholds in detecting patients at risk of mid-term PSCI. Furthermore, we identified an optimal demographically adjusted MoCA score predictive of mid-term PSCI to be used in the acute stroke setting.

Our results show that the categorization of acute stroke patients into normal/impaired based on thresholds proposed for MoCA by normative datasets is not recommended. The different thresholds produced a variability of approximately 13% in rates of patients classified as having cognitive deficits in the acute phase. Even more relevant, the general population normality thresholds underestimated patients at risk of a persistent cognitive decline, and about half of patients could miss the opportunity to benefit from cognitive rehabilitation or other therapeutic strategies. However, demographically adjusted MoCA scores were able to weigh the risk of PSCI, and this allowed us to identify a new predictive score. A good level of diagnostic accuracy was reached applying the correction norms by Aiello and colleagues, and a demographically adjusted MoCA predictive score of 22.8 points appears recommendable in the acute stroke setting. The higher accuracy of the Aiello’s dataset in predicting PSCI is likely due to methodological and cultural factors. From the methodological point of view, the Aiello’s dataset is the most recent and is based on the largest sample, and this could have probably increased its representativeness of the population. On the other side, the relevance of region-specific norms is an emerging issue in reliability of normative datasets, and the Aiello’s datasets, which was collected in Northern Italy, could be more consistent also from the cultural point of view with our sample from Central-Northern Italy.

Despite the availability of several studies that evaluated the use of MoCA in the acute phase of stroke [38–46], data on its predictivity on mid- and long-term PSCI diagnosis reached by means of a comprehensive neuropsychological battery are quite limited [22, 47, 48]. Furthermore, previous studies estimated MoCA predictive scores in acute stroke based on raw scores and did not calculate correction norms based on demographics. Since performance on neuropsychological tests is known to vary as a function of several demographic variables, and available normative datasets confirmed these effects also for MoCA, normality thresholds based on raw scores could be inaccurate [49]. Moreover, also the heterogeneity among MoCA normality cut-offs found in previous studies in acute stroke patients could be partly due to the neglected cultural and demographic differences among populations. In line with this, a recent study on optimal MoCA cut-off scores for people with probable Alzheimer’s disease confirmed the discrepancy in cut-off points existing between Italian and other international validation studies and debated the influence of specific population characteristics [50]. Finally, compared with previous evidence, we found a higher MoCA normality threshold, and this discrepancy is likely due to the fact that demographic corrections overall increased MoCA scores in an elderly cohort.

Our sample also included a small group of TIA patients. When the analyses were repeated excluding this subgroup of patients, the main results (i.e., variability in rates of patients having an impaired MoCA performance, inadequate accuracy of the normative thresholds, and superiority of the Aiello’s dataset in predicting PSCI) were confirmed (data nor shown). Considering that the distinction between TIA and stroke is often not easy because many TIA patients develop indeed acute brain lesions, and also because this distinction is somehow questionable, we decided to keep this small number of TIA patients in our sample [51].

Limitations of our study need to be highlighted. First, neuropsychological tests used at the follow-up evaluation in the two centers were not completely superimposable. Despite the two neuropsychological batteries assessed all core cognitive domains and were partially overlapping, the Luigi Sacco University Hospital protocol was more comprehensive and strengthened the evaluation of specific cortical deficits, such as aphasia and neglect. However, it should be noted that, despite these differences, the rate of PSCI was similar between the two centers. A second limitation is the high number of drop-outs that resulted in a limited sample size. From the statistical point of view, these limitations have reduced the statistical power, and our results need to be taken with some caution and further tested in larger studies. On the other side, this could also represent a selection bias that might have underestimated the PSCI rate considering that patients lost to follow-up presented worst baseline cognitive efficiency. From the clinical point of view, patients with severe cognitive impairment in the acute phase are, in most cases, already addressed to rehabilitation or long-term care pathways, while mild stroke patients are frequently discharged at home, thus increasing the risk that persistent cognitive deficits are underdiagnosed and, ultimately, undertreated. This is why tools able to screen for these patients in the acute phase may be useful.

Further research efforts are needed for the determination of the optimal MoCA predictive score in the acute setting. National longitudinal studies based on large samples of acute stroke patients should be conducted in order to provide corrections norms and predictive scores that could be appropriate from both cultural and clinical points of view.

## Data Availability

The datasets generated during and/or analyzed during the current study are available from the corresponding author on reasonable request.
